# Supplementary material for: Causes and MEchanisms foR non-atopic Asthma in Children (CAMERA) study: rationale and protocol
Source: Respir Res. 2025 Jun 5;26:212. doi: 10.1186/s12931-025-03279-6 (PMC12142945; doi:10.1186/s12931-025-03279-6)
Supplement: Supplementary file 1 — Additional file 1: Map showing the location of CAMERA study centres by asthma prevalence. [file 12931_2025_3279_MOESM1_ESM.docx]

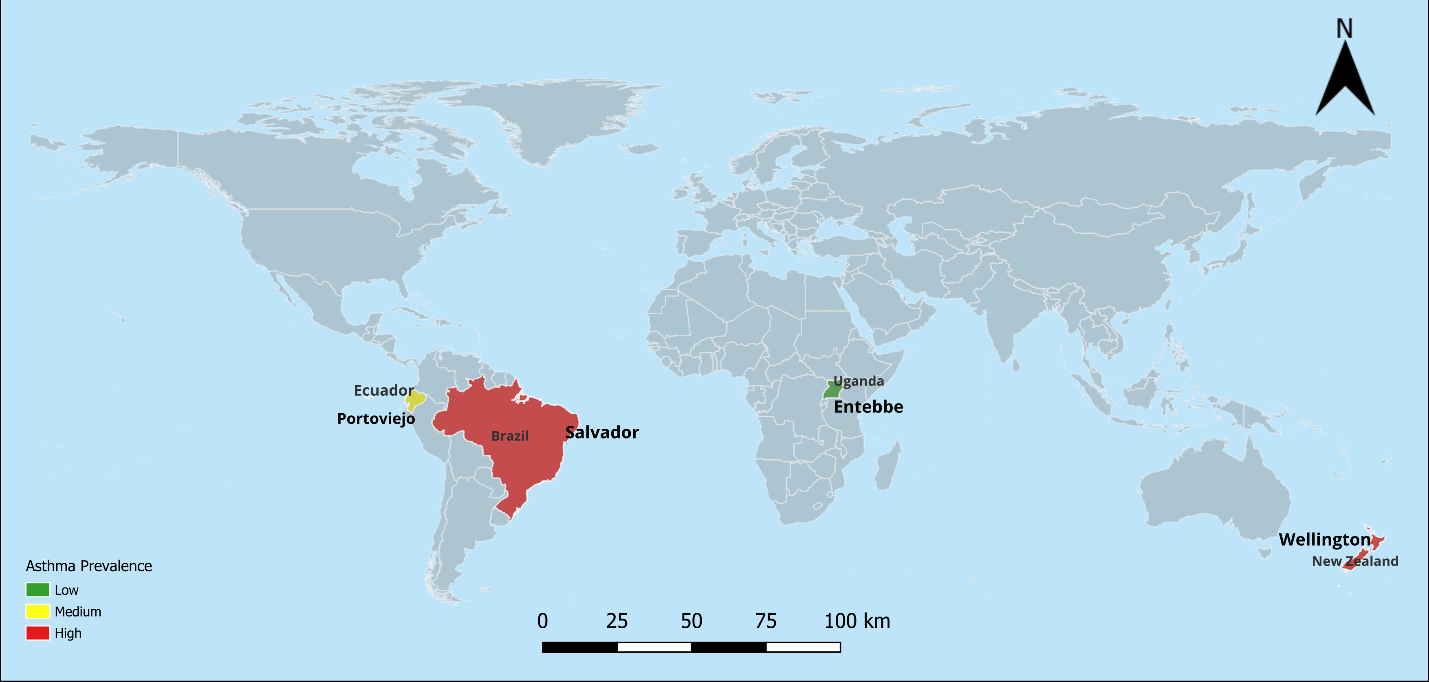


Figure S1: Map showing the location of CAMERA study centres by asthma prevalence

^*Brazil, Ecuador, and Uganda are LMICs whereas New Zealand is a HIC^
