# Supplementary material for: Causes and MEchanisms foR non-atopic Asthma in Children (CAMERA) study: rationale and protocol
Source: Respir Res. 2025 Jun 5;26:212. doi: 10.1186/s12931-025-03279-6 (PMC12142945; doi:10.1186/s12931-025-03279-6)
Supplement: Supplementary file 2 — Additional file 2: Skin prick test allergens to be used in each study centre. [file 12931_2025_3279_MOESM2_ESM.docx]

Table S1:Skin prick test allergens to be used in each study centre

| **Allergen list** | **Brazil** | **New Zealand** | **Ecuador** | **Uganda** |
| --- | --- | --- | --- | --- |
| (1) Negative control (saline)^*^ | √ | √ | √ | √ |
| (2) Grass mix |  | √ |  |  |
| (3) House dust mite (*Dermatophagoides pteronyssinus*)^*^ | √ | √ | √ | √ |
| (4) Cat^*^ | √ | √ | √ | √ |
| (5) Dog^*^ | √ | √ | √ | √ |
| (6) Mixed tree pollen^*^ | √ | √ | √ | √ |
| (7) House dust mite (*Dermatophagoides farinae*) | √ |  | √ |  |
| (8) House dust mite (*Dermatophagoides* mix) |  |  |  | √ |
| (9) *Cladosporium herbarum* (mould) |  |  | √ |  |
| (10) *Aspergillus fumigatus* (mould) |  |  | √ |  |
| (11) Weed mix |  |  |  | √ |
| (12) *Alternaria alternata* (mould mix) |  |  |  | √ |
| (13) Feather mix |  |  |  | √ |
| (14) *Blomia tropicalis* | √ |  | √ | √ |
| (15) *Blatela germanica* (cockroach) | √ |  | √ |  |
| (16) *Periplaneta Americana* (cockroach) | √ |  | √ | √ |
| (17) Positive control (histamine)^*^ | √ | √ | √ | √ |

^*Core allergens to be test^
